# Seroepidemiological Studies of Crimean-Congo Hemorrhagic Fever Virus in Domestic and Wild Animals

**DOI:** 10.1371/journal.pntd.0004210

**Published:** 2016-01-07

**Authors:** Jessica R. Spengler, Éric Bergeron, Pierre E. Rollin

**Affiliations:** Viral Special Pathogens Branch, Division of High Consequence Pathogens and Pathology, National Center for Emerging and Zoonotic Infectious Diseases, Centers for Disease Control and Prevention, Atlanta, Georgia, United States of America; University of Queensland, AUSTRALIA

## Abstract

Crimean-Congo hemorrhagic fever (CCHF) is a widely distributed, tick-borne viral disease. Humans are the only species known to develop illness after CCHF virus (CCHFV) infection, characterized by a nonspecific febrile illness that can progress to severe, often fatal, hemorrhagic disease. A variety of animals may serve as asymptomatic reservoirs of CCHFV in an endemic cycle of transmission. Seroepidemiological studies have been instrumental in elucidating CCHFV reservoirs and in determining endemic foci of viral transmission. Herein, we review over 50 years of CCHFV seroepidemiological studies in domestic and wild animals. This review highlights the role of livestock in the maintenance and transmission of CCHFV, and provides a detailed summary of seroepidemiological studies of wild animal species, reflecting their relative roles in CCHFV ecology.

## Introduction

Crimean-Congo hemorrhagic fever virus (CCHFV), a nairovirus of the *Bunyaviridae* family, is the causative agent of a severe human hemorrhagic fever disease characterized by fever, weakness, myalgia, and hemorrhagic signs [1]. Clinical disease is restricted to humans and is fatal in 3%–30% of cases. Crimean-Congo hemorrhagic fever (CCHF) has been described over a wide geographic area including Asia, Africa, and Europe. The natural vector and reservoir has been identified as *Hyalomma* spp. ticks, and the distribution of human cases closely mirrors vector distribution. CCHFV is transmitted to humans by the bite of an infected tick, contact with patients during the acute phase of illness, or by contact with blood or tissues of viremic animals. Early diagnosis is critical for patient support and for preventing spread of infection through well-documented human-to-human transmission [2]. Ribavirin has been used extensively as an antiviral treatment, but remains controversial [3,4].

In general, CCHFV circulates in nature in unnoticed enzootic tick–vertebrate–tick cycles. Asymptomatic CCHFV infection has been reported in numerous vertebrate species and appears to be pervasive in both wild and domestic animals [5]. Asymptomatic viremia lasting up to 7–15 days has been described in several vertebrate animal species [6–8], and CCHFV has been isolated from livestock and small mammals. An extensive amount of research has been conducted on CCHFV reservoir species and their respective roles in virus maintenance and transmission. Seroepidemiological studies comprise the majority of this research, elucidating reservoir species and virus circulation. CCHFV serosurveillance has relied on a variety of techniques, including virus neutralization assays [9,10], reverse passive hemagglutination inhibition (RPHI) assays [11–13], immunodiffusion assays such as agar gel diffusion precipitation (AGDP) [14,15], complement fixation (CF) assays [9,16–18], indirect immunofluorescence assays (IFA) [19–23], indirect or sandwich enzyme-linked immunoassays (ELISA) [23–27], and competitive ELISA (CELISA) [28].

Several groups have published reports of detailed serosurveys conducted recently in various countries, including Albania, Iran, Sudan, and India. However, numerous studies investigating serological evidence of CCHFV in animal species were performed decades ago, are difficult to obtain, and are often published in non-English languages. Animal serosurvey data have been examined and discussed in CCHFV reviews [1,6], but no literature currently exists cohesively presenting current and past reports of the presence or absence of CCHFV antibodies in domestic and wild animals. Virus emergence and reemergence continue to be key topics of national and international health security. As with other hemorrhagic fever viruses, the potential introduction of CCHFV into new geographic areas [29–31] should be considered and requires appropriate knowledge of virus ecology, transmission dynamics, and competent reservoir hosts and vectors.

**Fig 1:**
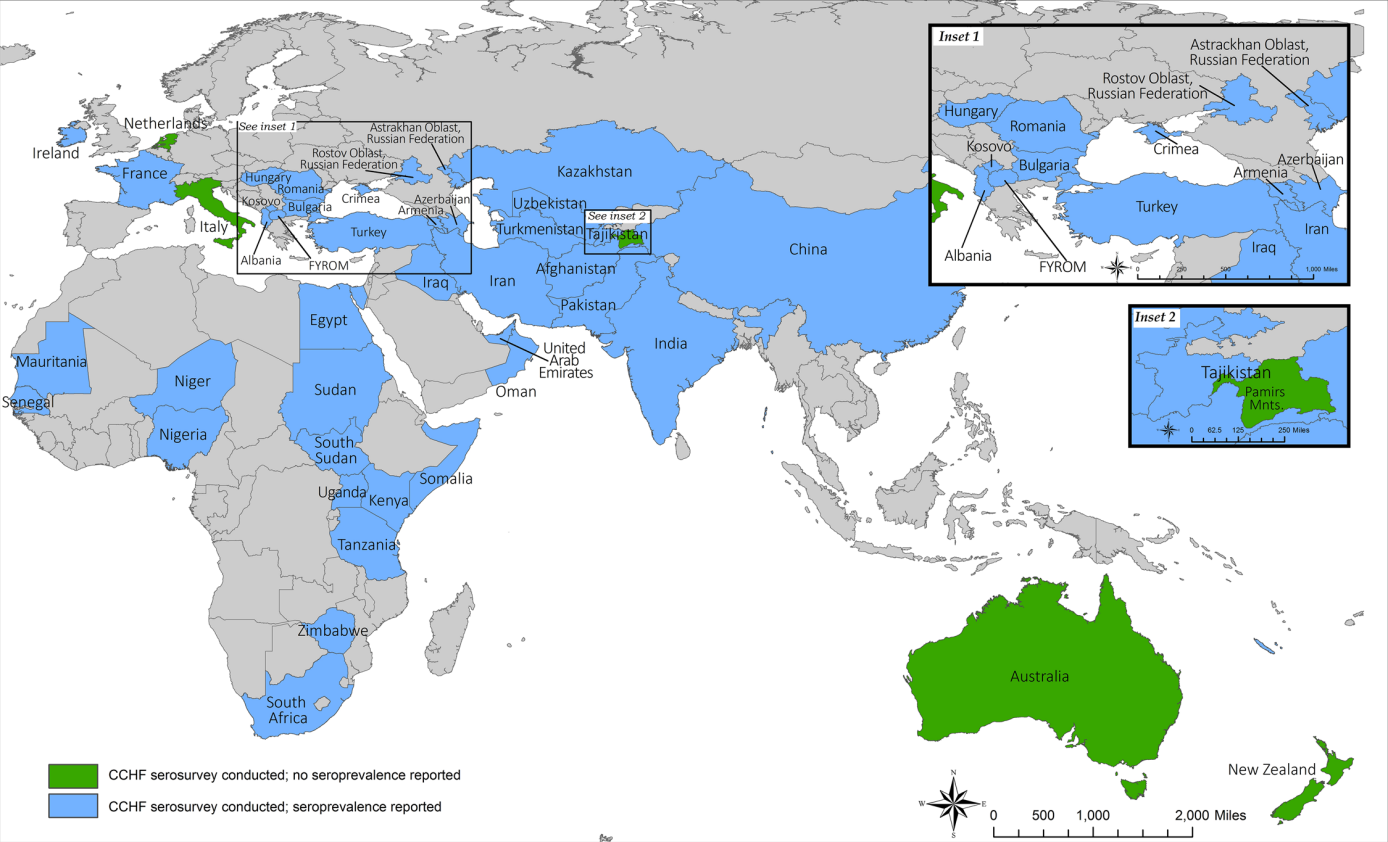
Geographic summary of countries represented in CCHFV seroepidemiological surveys. Countries with evidence of seroprevalence in animals represented in blue, countries with absence of seroprevalence represented in green, and countries without reported serosurveys represented in grey.

Herein, we provide a detailed summary of the extensive seroepidemiological CCHFV studies performed internationally in both domestic and wild animals (Fig 1). This report serves as an important resource in discussion of the role of animals in CCHFV maintenance and transmission to humans. The information provided specifically aids in understanding the global impact of CCHFV and clarifying the roles of domestic and wild animals in putative expansion of CCHFV endemic regions.

## Domestic Animals

**Fig 2:**
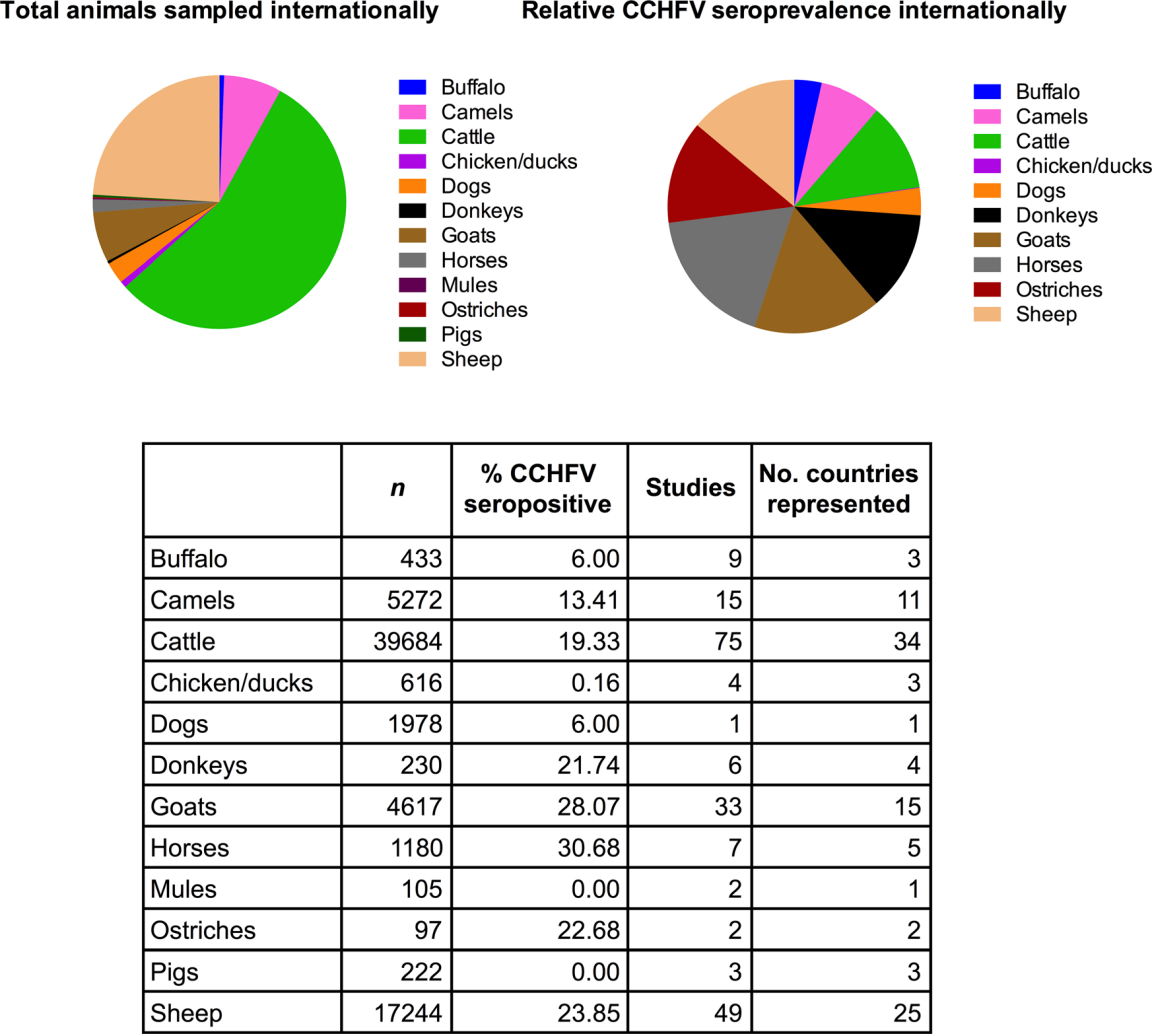
Total international CCHFV seroprevalence reported in domestic animals by species. Seroprevalence determined by sum of seropositive animals over the sum of total animals, sampled internationally. Studies that did not report sample numbers or differentiate between types of animal were excluded.

Seroepidemiological studies in endemic regions indicate that various domestic and peri-domestic animals could be asymptomatically infected with CCHFV. Detection of CCHFV antibodies in domestic animals has been important in providing initial evidence of circulating virus and in localizing CCHFV foci and increased risk for human infection [6,32,33]. A wide spectrum of domestic animal species has been investigated internationally, including cattle, sheep, goats, horses, pigs, dogs, and chickens (Table 1). Other domestic species investigated include buffalo, camels, and ostriches. Examples of high seroprevalence in domestic animals include 79.1% seropositive cattle (Afghanistan) [34], 75.0% sheep (Afghanistan) [34], 66.0% goats (Turkey) [10], 58.8% horses (Iraq) [35], and 39.5% donkeys (Tajikistan) [36]. High seroprevalence has also been reported in camels; the highest (excluding the 1/1 animal found positive in Pakistan) percentage of seropositive camels was reported in Kenya at 26% (*n* = 499). The largest reported sample size of a single species comprised almost 9,000 cattle tested in South Africa [37]. The role of cattle, sheep, and other large vertebrates in CCHFV ecology is reflected in the relative levels of species-specific CCHFV antibody prevalence reported internationally (Fig 2). Among studies that indicate sample size, cattle are the most often studied (75 studies), followed by sheep (49 studies) and goats (33 studies). Data on cattle and sheep have also been reported from the largest number of countries (34 and 25, respectively) (Table 1). Reports of other species are more limited; seroprevalence in domestic dogs, for example, was only reported in one study based on samples obtained in South Africa and Zimbabwe [13].

**Table 1:** Crimean-Congo hemorrhagic fever virus (CCHFV) seroprevalence in domestic animals.

Animal	Country (Region) of Specimen Origin	Seroprevalence		Assay	Reference
		*n*	*%*		
**Buffalo**	Egypt	47	0	CF	[38]
	Egypt (central)	153	0	IgG ELISA	[39]
	India	2	0	AGDP	[18]
	India (Jammu and Kashmir)	23	0	CF	[40]
	India (Jammu and Kashmir)	14	0	AGDP	[40]
	India	3	0	IgG ELISA	[41]
	India (Maharashtra, Rajasthan)	46	2.2	IgG ELISA	[42]
	India (Ahmadabad)	123	19.5	IgG ELISA	[42]
	Pakistan	22	4.5	CF	[43]
**Camels**	China (Tarim, Junggar, and Turpan-Hami Basins)	10	40	RPHI	[44]*
	Egypt	34	8.8	CF	[38]
	Egypt (central)	10	0	IgG ELISA	[39]
	India (Jammu and Kashmir)	3	0	CF	[40]
	India (Jammu and Kashmir)	3	0	AGDP	[40]
	Iran	99	19.1	AGDP	[45]
	Iran	157	0	AGDP	[9] in [6]
	Iraq	99	23.2	CF	[35]
	Kenya	499	26	AGDP, IFA	[20]
	Niger	353	13.6	IgG ELISA	[21]
	Oman	109	16	IgG ELISA	[46]
	Pakistan	1	100	IgG ELISA	[47]
	Russia (Astrakhan Oblast)	NR	1.4	AGDP	[48] in [6]
	Sudan	3802	12	AGDP, IFA	[20]
	Sudan	13	7.7	IgG ELISA	[47]
	United Arab Emirates	80	6.3	IgG ELISA	[47]
**Cattle**	Afghanistan	230	5.6	AGDP	[18,49]
	Afghanistan (Engil District)	92	79.1	IgG ELISA	[34]
	Albania	14	0	IgG ELISA	[50]
	Albania (ten regions surveyed)	337	4.74	IgG ELISA	[51]
	Albania (Berat)	50	4	IgG ELISA	[52]
	Albania (Gjirokastra)	50	2.1	IgG ELISA	[53]
	Albania (Kolonje)	54	7.4	IgG ELISA	[52]
	Albania (Kukes)	11	0	IgG ELISA	[53]
	Albania (Rreshen)	40	2.6	IgG ELISA	[53]
	Armenia	1373	4.2	AGDP	[54]
	Azerbaijan (Sabirabad and Saatly)	651	16.2	AGDP	[55]
	Azerbaijan (Sal’yany)	142	11.9	AGDP	[55]
	Azerbaijan (Pushkino)	38	10.1	AGDP	[55]
	Azerbaijan (Apsheron)	102	11.0	AGDP	[55]
	Azerbaijan (Divichin)	161	3.1	AGDP	[55]
	Azerbaijan (Lenkoran’)	238	3.8	AGDP	[55]
	Azerbaijan (Sabirabad)	454	4.2	AGDP	[55]
	Azerbaijan (Saatly)	424	4.7	AGDP	[55]
	Bulgaria	1756	33.2	AGDP	[56]
	Bulgaria (Municipality of Aytos)	127	71	IgG ELISA	[27]
	Bulgaria	1775	7.89	IFA	[22]
	Egypt	43	0	CF	[38]
	Egypt	200	0	AGDP, IFA	[20]
	Egypt (central)	161	0.6	IgG ELISA	[39]
	Germany	78	0	RPHI	[57]
	Hungary	687	0.9	AGDP	[58]
	Hungary (Hajdú-Bihar)	161	0	AGDP	[59]
	India	22	0	AGDP	[15]
	India	25	0	AGDP	[18]
	India	12	0	AGDP	[18]
	India	711	12.1	IgG ELISA	[60]
	India	32	43.8	IgG ELISA	[41]
	India (northern West Bengal)	5	0	IgG ELISA	[42]
	India (Ahmadabad)	74	4.1	IgG ELISA	[42]
	India (Jammu and Kashmir)	66	0	CF	[40]
	India (Jammu and Kashmir)	55	0	AGDP	[40]
	Iran	100	19	AGDP	[45]
	Iran	130	18	AGDP	[9]
	Iran	876	5.9	ELISA	[61]
	Iran (Ardabil Province)	10	30	IgG ELISA	[62]
	Iran (Isfahan Province)	15	20	IgG ELISA	[63]
	Iran	1091	25.0	IgG ELISA	[64]
	Iraq	411	29.3	CF	[35]
	Iraq (Basrah, southern Iraq)	48	37	IgG ELISA	[65]
	Ireland	54	1.9	RPHI	[57]
	Italy	50	0	RPHI	[57]
	Kazakhstan	842	0.7	AGDP	[66]
	Kenya/Uganda	93	76.3	AGDP	[45]
	Kosovo	353	18.4	IgG ELISA	[67]
	Niger	1201	46	IgG ELISA	[21]
	Nigeria	1164	25.7	AGDP	[68]
	Oman	27	4	IgG ELISA	[46]
	Pakistan	45	2.2	CF	[43]
	Pakistan	1	0	IgG ELISA	[47]
	Pamirs	189	0	AGDP	[36]
	Republic of Macedonia	158	14.6	IgG ELISA	[24]
	Russia (Astrakhan Oblast)	NR	5.1	CF, AGDP	[69]
	Russia (Rostov Oblast)	430	23.0	AGDP	[70]
	Russia (Rostov Oblast)	355	2.8	AGDP	[71]
	Russia (Rostov Oblast)	2155	0.5–17.0	AGDP	[72]
	Senegal	1269	6.1	AGDP	[73]
	Somalia	16	6.3	IgG ELISA	[47]
	South Africa	8667	28	RPHI	[37]
	South Africa	6128	26.5	RPHI	[74]
	Sudan (North Kurdufan State)	299	7.0	IgG ELISA	[75]
	Sudan (East Darfur State)	282	19.14	IgG ELISA	[26]
	Tajikistan (FRM Tajik SSR, northern)	184	0	AGDP	[36]
	Tajikistan (FRM Tajik SSR)	1585	1.1	AGDP	[36]
	Tajikistan (FRM Tajik SSR)	775	1.1	AGDP	[76]
	Turkmenistan (FRM Turkmen SSR, Ashkhada region)	199	3.5	AGDP	[32]
	Turkmenistan (FRM Turkmen SSR, Geok-Tepe region)	29	31	AGDP	[32]
	Tanzania (central zone: Mpwapwa)	166	0.6	AGDP	[6]
	Tanzania (northern zone: Longido, Monduli, Tengeru)	256	7.4	AGDP	[6]
	Tanzania (Sukumaland)	209	4.8	AGDP	[6]
	Tanzania (Lake Victoria coastal region)	417	6.3	AGDP	[6]
	Turkey (Marmara region)	201	13	IgG ELISA	[10]
	The Netherlands	7	0	IgG ELISA	[47]
	Uganda	104	36.5	AGDP	[77]
	United Arab Emirates	34	0	IgG ELISA	[47]
	Zimbabwe	763	45	RPHI	[37]
**Chickens**	Kosovo	8	0	IgG ELISA	[67]
	Tajikistan (FRM Tajik SSR)	136	0	CF, AGDP	[76]
**Chickens/ducks****†**	Kazakhstan	428	0.2	AGDP	[66]
**Dogs**	South Africa, Zimbabwe	1978	6	RPHI	[13]
**Donkeys**	Azerbaijan (Sal’yany)	69	18.8	AGDP	[55]
	Bulgaria	103	17.4	AGDP	[56]
	Bulgaria (Municipality of Aytos)	8	50	IgG ELISA	[27]
	India (Jammu and Kashmir)	6	0	CF	[40]
	India (Jammu and Kashmir)	6	0	AGDP	[40]
	Tajikistan (FRM Tajik SSR)	38	39.5	AGDP	[36]
**Ducks**	Tajikistan (FRM Tajik SSR)	44	0	CF, AGDP	[76]
**Goats**	Afghanistan	233	9	AGDP	[18,49]
	Albania	10	20	IgG ELISA	[50]
	Bulgaria	411	62.3	AGDP	[56]
	Bulgaria (Municipality of Aytos)	15	60	IgG ELISA	[27]
	India	1	0	IgG ELISA	[47]
	India	117	9.4	AGDP	[15]
	India	45	40	AGDP	[15]
	India	186	16.1	AGDP	[18]
	India	279	41.2	IgG ELISA	[60]
	India	28	46.4	IgG ELISA	[41]
	India (Maharashtra, northern West Bengal, Rajasthan)	146	2.1	IgG ELISA	[42]
	India (Ahmadabad)	76	30.3	IgG ELISA	[42]
	India (Jammu and Kashmir)	75	0	CF	[40]
	India (Jammu and Kashmir)	35	0	AGDP	[40]
	Iran	135	36	AGDP	[9]
	Iran	5	40	IgG ELISA	[47]
	Iran (Ardabil Province)	3	33.3	IgG ELISA	[62]
	Iran (Khorasan Province)	150	46	NR	In [63]
	Iran (Isfahan Province)	21	9.5	IgG ELISA	[63]
	Iran	987	24.8	IgG ELISA	[64]
	Iraq	562	49.6	CF	[35]
	Kosovo	10	10	IgG ELISA	[67]
	Mauritania	27	11.1	IgG ELISA	[78]
	Mauritania	27	0	IgM ELISA	[78]
	Niger	224	4.9	IgG ELISA	[21]
	Oman	146	14	IgG ELISA	[46]
	Pakistan	48	0	CF	[43]
	Pakistan	1	0	IgG ELISA	[47]
	Somalia	14	21.4	IgG ELISA	[47]
	Sudan	356	3.9	RPHI	[57]
	Turkey	76	0	RPHI	[57]
	Turkey (Marmara region)	147	66.0	IgG ELISA	[10]
	United Arab Emirates	21	0	IgG ELISA	[47]
**Goats/sheep†**	Iran	201	45	AGDP	[45]
	Kazakhstan	832	0.4	AGDP	[66]
	Tajikistan (FRM Tajik SSR, central)	107	0.9	AGDP	[36]
	Tajikistan (FRM Tajik SSR)	326	1.5	AGDP	[76]
	Turkmenistan (FRM Turkmen SSR)	663	11.3	AGDP	[32]
**Horses**	Bulgaria	536	39	AGDP	[56]
	Hungary (Hajdú-Bihar)	8	0	AGDP	[59]
	India	282	1.1	AGDP	[18]
	India (Jammu and Kashmir)	16	0	CF	[40]
	India (Jammu and Kashmir)	15	0	AGDP	[40]
	Iraq	252	58.8	CF	[35]
	Russia (Astrakhan Oblast)	NR	3.1	CF, AGDP	[69]
	Russia (Rostov Oblast)	NR	Pos	AGDP	[70]
	Tajikistan (FRM Tajik SSR)	71	2.8	AGDP	[76]
**Misc. small ruminants/livestock†**	Iran (Isfahan Province)	NR	56	NR	In [63]
	Kosovo (excluding sheep)	NR	14	IgG ELISA	[79]
	Niger	418	10.3	IgG ELISA	[21]
	Senegal	1269	6.1	AGDP	[73]
**Misc. domestic animals†**	India	40	2.5	AGDP	[18]
	India	139	7.9	AGDP	[18]
**Mules**	India (Jammu and Kashmir)	64	0	CF	[40]
	India (Jammu and Kashmir)	41	0	AGDP	[40]
**Ostriches**	Iran	5	20	IgG ELISA	[80]
	South Africa	92	23.9	RPHI	[17]
**Pigs**	Egypt	46	0	CF	[38]
	India (Maharashtra)	25	0	IgG ELISA	[42]
	Russia (Rostov Oblast)	151	0	AGDP	[72]
**Sheep**	Afghanistan (Engil District)	40	75.0	IgG ELISA	[34]
	Azerbaijan (Sabirabad and Saatly)	91	16.2	AGDP	[55]
	Azerbaijan (Pushkino)	89	6.7	AGDP	[55]
	Australia	30	0	IgG ELISA	[47]
	Bulgaria	1190	32.9	AGDP	[56]
	Bulgaria (Municipality of Aytos)	242	74	IgG ELISA	[27]
	China (Tarim, Junggar, and Turpan-Hami basins)	3640	12.6	RPHI	[44]*
	Egypt	52	23.1	CF	[38]
	Egypt	400	0	AGDP, IFA	[20]
	Egypt (central)	174	0	IgG ELISA	[39]
	Greece (Kastoria)	40	25.0	IgG ELISA	[81]
	Hungary	48	31.3	AGDP	[58]
	India	13	7.7	AGDP	[15]
	India	136	0	AGDP	[18]
	India	149	0.7	AGDP	[18]
	India	236	32.6	IgG ELISA	[60]
	India	19	47.4	IgG ELISA	[41]
	India (Maharashtra, Rajasthan)	17	35.3	IgG ELISA	[42]
	India (Ahmadabad)	32	50	IgG ELISA	[42]
	India (Jammu and Kashmir)	38	0	CF	[40]
	India (Jammu and Kashmir)	12	0	AGDP	[40]
	Iran	728	38	AGDP	[9]
	Iran	2	0	IgG ELISA	[47]
	Iran (Ardabil Province)	43	41.9	IgG ELISA	[62]
	Iran (Mazandaran Province)	270	3.7	IgG ELISA	[82]
	Iran (Khorasan Province)	298	77.5	NR	In [63]
	Iran (Isfahan Province)	286	12.6	IgG ELISA	[63]
	Iran	2447	58.7	IgG ELISA	[64]
	Iraq	769	57.6	CF	[35]
	Iraq (Basrah, southern Iraq)	74	20	IgG ELISA	[65]
	Kosovo	30	10	IgG ELISA	[67]
	Kosovo	NR	32.6	IgG ELISA	[79]
	Mauritania	70	20	IgG ELISA	[78]
	Mauritania	70	0	IgM ELISA	[78]
	New Zealand	67	0	RPHI	[57]
	Niger	271	3	IgG ELISA	[21]
	Oman	34	3	IgG ELISA	[46]
	Pakistan	46	0	CF	[43]
	Pamirs	266	0	AGDP	[36]
	Romania (Tulcea, northern Dobrogea)	471	27.8	IgG ELISA	[33]
	Russia (Astrakhan Oblast)	NR	0.3	CF, AGDP	[69]
	Senegal	942	10.4	IgG ELISA	[83]
	Somalia	12	50	IgG ELISA	[47]
	Somalia	28	0	RPHI	[57]
	Sudan	1972	4.3	RPHI	[57]
	Tajikistan	614	2.6	AGDP	[36]
	Tajikistan (northern)	379	0	AGDP	[36]
	Tajikistan (central)	82	4.9	AGDP	[36]
	Turkey	95	3.2	RPHI	[57]
	Turkey (Marmara region)	160	31.8	IgG ELISA	[10]
	United Arab Emirates	30	0	IgG ELISA	[47]

NR, not reported; FRM, formerly; SSR, Socialist Soviet Republic; AGDP, agar gel diffusion precipitation; CF, complement fixation; ELISA, enzyme linked immunosorbent assay; IFA, immunofluorescence assay; Pos, seropositivity reported; RPHI, reverse passive hemagglutination inhibition assay.

*Personal communication with Drs. Zhihong Hu and Yujiang Zhang for species breakdown of sample count

†Sample numbers and results not differentiated by animal.

Domestic animal species are often implicated in CCHFV transmission when human CCHF cases are detected. Sheep have been recognized as very important CCHFV reservoirs in certain endemic regions, and have been epidemiologically linked to human cases on several occasions [64,79,84,85]. In Uzbekistan, three CCHF cases were described in persons involved in the handling of tissue from a cow [86]. Similarly, the first patient in an epizootic of CCHFV in Mauritania became ill shortly after butchering a goat [78]. As such, increased CCHFV IgG seropositivity in livestock often parallels reports of CCHF cases in humans with exposure to livestock (e.g., slaughterers, butchers, and farmers), particularly in those who handle blood and organs from infected livestock [34,87–92]. Conversely, negative seroprevalence results in domestic animal samples reflect either low-level transmission or the absence of CCHFV in those geographic areas. Thus, no evidence of seroprevalence in domestic animals was found in samples from Germany, Italy, the Netherlands, Australia, or New Zealand, all countries with no CCHFV cases reported to date [57].

The tick–vertebrate–tick cycle of CCHFV maintenance is reflected in relative tick abundance and associated animal seroprevalence. Cattle heavily infested with ticks were more likely to be CCHFV seropositive [26,75], and vector control to reduce the tick burden was associated with decreased seroprevalence [75]. Cattle are noted as the most sensitive indicator of low-level CCHFV circulation because they tend to be highly infested with *Hyalomma* spp. ticks, the numbers of which can be ten times higher than those found on small ruminants [93]. In Iran, following detection of human CCHFV cases in Kurdistan Province in 2007, ticks were collected from cattle, sheep, and goats. Of the collected ticks, 5.6% (5/90) were positive by reverse transcription PCR for CCHFV, and four of the five positive ticks were collected from cattle [94]. While there appears to be an association between the presence of infected ticks and detection of seropositive animals [95], viral RNA in attached ticks does not directly indicate seropositivity in host species, and vice versa: infected ticks have been found on seronegative animals and uninfected ticks on seropositive animals.

Abiotic variation by season, country, and region is reported in CCHFV seroprevalence studies. Studies in Turkmenistan (then Turkmen Soviet Socialist Republic [SSR]) reported an increase in CCHFV seropositive domestic animal species during the summer season, and found large variations between regions and individual farms (seropositivity range 5.9%–32%) [32]. Geographic variation of CCHFV seroprevalence in domestic animals within a single country has also been reported in several studies [10,51,61,82]. Longitudinal studies in Russia (Rostov Oblast) demonstrated considerable variation when repeated sampling was performed in the same location. These studies reported September as the optimum period for detecting precipitating antibodies in this area, with a notable decrease in seroprevalence in the winter–spring period [71]. In support of the recognized endemic transmission cycle of CCHF, variation in seroprevalence is often associated with competent vector distribution, host preference of competent tick vectors, and tick load on a particular animal species. Anti-CCHFV antibody prevalence is highest in biotopes where *Hyalomma* spp. ticks often predominate. Sustained endemic transmission is found only where *Hyalomma* spp. ticks are present, and epizootic transmission occurs during periods of increased abundance of these ticks [96]. In the hyperendemic CCHFV region in Turkey, the overall tick infestation rate of livestock was 61.2%; 63.1% of cattle and 56.9% of sheep were infested with one or more tick. The dominant species infesting both cattle and sheep was *Hyalomma marginatum* [97].

A subset of biotic factors determining domestic animal CCHFV seroprevalence were investigated in Senegalese sheep by Wilson et al., who reported that the sex of the animal did not affect antibody prevalence [83]. Other factors, including increasing age, are consistently associated with higher seroprevalence in domestic animals [26,27,61,75,82]. Age likely reflects repeated exposure potential, as described by Adam et al., who found that calves started to get infected after the age of two, the age at which they are released to pasture for grazing and, thus, are more likely to be exposed to infected ticks [75]. Breed may also play a role: in Sudan, cross-bred cattle were 37 times more likely to be seropositive than endogenous breeds [75]. Further insight into the dynamics of infection in domestic species was provided by a longitudinal serosurvey conducted by Zeller et al. in Senegal from 1989 to 1992 [95]. Investigators collected ticks feeding on two cows and 12 goats, and obtained paired blood samples three times per month. Seropositive animals infested with infected ticks had even higher anti-CCHFV IgG antibody titers than seropositive animals without ticks, supporting the occurrence of reinfection in domestic species. The persistence of anti-CCHFV IgM antibodies in naturally infected animals was found to be 1–2 months [95].

Studies in companion animals are very limited and, thus, difficult to broadly interpret. Antibodies to CCHFV were reported in 6% (*n* = 1978) of dogs in South Africa and Zimbabwe [13]. In another study, in association with human CCHF cases in Mauritania in 2003, feeding ticks were collected from livestock and dogs. A proportion (five of 56 tested) of *Rhipicephalus evertsi evertsi* ticks collected from sheep were found to be CCHFV positive by reverse transcription PCR, but none of the five *Rhipicephalus sanguineus* ticks collected from dogs were positive [78]. While vector competence and host preference may indicate the risk of natural infection and transmission in companion animal species in the absence of serological data, broadly translating vector data to risk of exposure remains complex, as tick data is not always consistent and is influenced by many factors unrelated to the host. For example, CCHFV has been isolated from *Rhipicephalus* spp. ticks [98]. However, *R*. *sanguineus* (brown dog ticks) have been reported as positive or negative for CCHFV depending on the study [44,99]. Additional data on companion animals and associated vector species will aid in more clearly evaluating the role of companion animals in the ecology of CCHFV.

## Wild Animals

The seroepidemiological reports of CCHFV in wild animals reviewed herein comprise almost 7,000 samples from over 175 avian, mammalian, and reptilian species (Table 2). Considerable seroprevalence was consistently reported in hares (3%–22%), buffalo (10%–20%), and rhinoceroses (40%–68%). Of the species investigated, those with low reported seroprevalence include elephants (single animal), marmots (no evidence), all non-human primate species (no evidence), and all insectivore rodent species (no evidence). While anti-CCHFV antibodies were not detected in Insectivora rodents, several seropositive hedgehogs (*Erinaceus europaeus*, *Hemiechinus auritus*) have been reported, and a substantial tick load of up to 40 larval and nymphal *H*. *marginatum* ticks has been described on hedgehog hosts during the peak season of immature tick activity [6,100]. However, the role of hedgehogs in enzootic maintenance appears to be variable by species. *H*. *auritus* develop viremia during experimental infection [101] and are considered a natural CCHFV reservoir by serving as a source of CCHFV for feeding ticks. In contrast, in the same study, experimental infection in the European hedgehog (*E*. *europaeus*) did not produce detectable viremia, suggesting reduced susceptibility to infection or more efficient viral clearance.

**Table 2:** CCHFV seroprevalence in wild animals.

Class	Order	Common name	Scientific name	Country (Region) of Specimen Origin	Seroprevalence		Test	Reference
					*n*	*%*		
N/A	N/A	Misc. wild animals	N/A	East Africa (Kenya, Uganda)	162	1.9	AGDP	[45]
Aves	Anseriformes	Common teal, Eurasian teal	*Anas crecca*	Turkmenistan (FRM Turkmen SSR, Gasan-Kuli region)	1	0	AGDP	[32]
Aves	Anseriformes	Red-billed teal	*Anas erythrorhyncha*	South Africa	9	0	RPHI	[17]
Aves	Anseriformes	Yellow-billed duck	*Anas undulata*	South Africa	91	0	RPHI	[17]
Aves	Apodiformes	Little swift	*Apus barbatus*	South Africa	15	0	RPHI	[17]
Aves	Charadriiformes	Caspian long-legged plover	*Charadrius* spp.	Turkmenistan (FRM Turkmen SSR, Gasan-Kuli region)	11	0	AGDP	[32]
Aves	Charadriiformes	Eurasian woodcock	*Scolopax rusticola*	Turkmenistan (FRM Turkmen SSR, Gasan-Kuli region)	1	0	AGDP	[32]
Aves	Charadriiformes	Gull	*Larus* spp.	Albania (Kukes)	6	0	IgG ELISA	[50]
Aves	Charadriiformes	Grey plover	*Pluvialis squatarola*	Turkmenistan (FRM Turkmen SSR, Gasan-Kuli region)	2	0	AGDP	[32]
Aves	Charadriiformes	Red-backed sandpiper, dunlin	*Erolia alpina*, *Calidris alpina*	Turkmenistan (FRM Turkmen SSR, Gasan-Kuli region)	4	0	AGDP	[32]
Aves	Charadriiformes	Redshank	*Tringa* spp.	Turkmenistan (FRM Turkmen SSR, Gasan-Kuli region)	5	0	AGDP	[32]
Aves	Charadriiformes	Sanderling	*Calidris alba*	Turkmenistan (FRM Turkmen SSR, Gasan-Kuli region)	4	0	AGDP	[32]
Aves	Charadriiformes	Snowy plover	*Charadrius nivosus*	Turkmenistan (FRM Turkmen SSR, Gasan-Kuli region)	10	0	AGDP	[32]
Aves	Ciconiiformes	Abdim’s stork	*Ciconia abdimii*	South Africa	7	0	RPHI	[17]
Aves	Columbiformes	Eurasian collared dove	*Streptopelia decaocto*	Albania (Kukes)	6	0	IgG ELISA	[50]
Aves	Columbiformes	European turtle dove	*Streptopelia turtur*	Albania (Kukes)	1	0	IgG ELISA	[50]
Aves	Columbiformes	Laughing dove	*Stigmatopelia senegalensis*	South Africa	14	0	RPHI	[17]
Aves	Columbiformes	Rock dove	*Columba livia*	Albania (Kukes)	6	0	IgG ELISA	[50]
Aves	Columbiformes	Pigeons/doves	*Columba* spp.	Albania (Kukes)	5	0	IgG ELISA	[50]
Aves	Galliformes	Helmeted guineafowl	*Numida meleagris*	South Africa	37	5‡	CELISA	[28]
Aves	Galliformes	Rock partridge	*Alectoris graeca*	Albania (Kukes)	3	0	IgG ELISA	[50]
Aves	Gruiformes	Common moorhen	*Gallinula chloropus meridionalis*	South Africa	13	0	RPHI	[17]
Aves	Misc. species	n/a	n/a	South Africa	32	0	RPHI	[17]
Aves	Passeriformes	Cape sparrow	*Passer melanurus melanurus*	South Africa	5	0	RPHI	[17]
Aves	Passeriformes	Eurasian magpie	*Pica pica*	Russia (Rostov Oblast)	NR	1 animal	AGDP, IHI	[102] in [6]
Aves	Passeriformes	Eurasian tree sparrow	*Passer montanus*	Albania (Kukes)	1	0	IgG ELISA	[50]
Aves	Passeriformes	Common starling	*Sturnus vulgaris*	Albania (Kukes)	1	0	IgG ELISA	[50]
Aves	Passeriformes	Hooded crow	*Corvus corone cornix*	Albania (Kukes)	5	0	IgG ELISA	[50]
Aves	Passeriformes	House sparrow	*Passer domesticus*	Albania (Kukes)	5	0	IgG ELISA	[50]
Aves	Passeriformes	South masked weaver	*Ploceus velatus inustus*	South Africa	16	0	RPHI	[17]
Aves	Passeriformes	Red bishop	*Euplectes orix*	South Africa	110	0	RPHI	[17]
Aves	Passeriformes	Red-billed quelea	*Quelea quelea*	South Africa	95	0	RPHI	[17]
Aves	Passeriformes	True thrushes	*Turdus* spp.	Albania (Kukes)	1	0	IgG ELISA	[50]
Aves	Passeriformes	Typical warblers	*Sylvia* spp.	Albania (Kukes)	1	0	IgG ELISA	[50]
Aves	Passeriformes	Woodchat shrike	*Lanius senator*	Albania (Kukes)	1	0	IgG ELISA	[50]
Aves	Pelecaniformes	Cattle egret	*Bubulcus ibis*	South Africa	39	0	RPHI	[17]
Aves	Pelecaniformes	African sacred ibis	*Threskiornis aethiopicus aethiopicus*	South Africa	14	0	RPHI	[17]
Aves	Struthioniformes	Ostrich	*Struthio camelus*	South Africa	9	0	CELISA	[28]
Mammalia	Artiodactyla	Black wildebeest	*Connochaetes gnou*	South Africa, Zimbabwe	30	0	RPHI	[13]
Mammalia	Artiodactyla	Blesbok	*Damaliscus dorcas*	South Africa, Zimbabwe	23	8.7	RPHI	[13]
Mammalia	Artiodactyla	Blue wildebeest	*Connochaetes taurinus*	South Africa, Zimbabwe	51	0	RPHI	[13]
Mammalia	Artiodactyla	Blue wildebeest	*Connochaetes taurinus*	South Africa	31	0	CELISA	[28]
Mammalia	Artiodactyla	Bushbuck	*Tragelaphus scriptus*	South Africa, Zimbabwe	8	0	RPHI	[13]
Mammalia	Artiodactyla	Bushbuck	*Tragelaphus scriptus*	South Africa	1	0	CELISA	[28]
Mammalia	Artiodactyla	Red river hog	*Potamochoerus porcus*	South Africa, Zimbabwe	3	0	RPHI	[13]
Mammalia	Artiodactyla	Duiker	*Sylvicapra grimmia*	South Africa, Zimbabwe	12	8.3	RPHI	[13]
Mammalia	Artiodactyla	Duiker	*Sylvicapra grimmia*	South Africa	1	0	CELISA	[28]
Mammalia	Artiodactyla	Common eland	*Taurotragus oryx*	South Africa, Zimbabwe	127	46	RPHI	[13]
Mammalia	Artiodactyla	Gemsbok	*Oryx gazella*	South Africa, Zimbabwe	13	46.2	RPHI	[13]
Mammalia	Artiodactyla	Giraffe	*Giraffa camelopardalis*	South Africa, Zimbabwe	3	100	RPHI	[13]
Mammalia	Artiodactyla	Giraffe	*Giraffa camelopardalis*	South Africa	44	23	CELISA	[28]
Mammalia	Artiodactyla	Grey rhebok	*Pelea capreolus*	South Africa, Zimbabwe	1	0	RPHI	[13]
Mammalia	Artiodactyla	Cape grysbok	*Raphicerus melanotis*	South Africa, Zimbabwe	13	0	RPHI	[13]
Mammalia	Artiodactyla	Lichtenstein’s hartebeest	*Sigmoceros lichtensteinii*	South Africa	1	0	CELISA	[28]
Mammalia	Artiodactyla	Hippopotamus	*Hippopotamus amphibius*	South Africa, Zimbabwe	6	0	RPHI	[13]
Mammalia	Artiodactyla	Hippopotamus	*Hippopotamus amphibius*	South Africa	15	0	CELISA	[28]
Mammalia	Artiodactyla	Impala	*Aepyceros melampus*	South Africa, Zimbabwe	211	1.4	RPHI	[13]
Mammalia	Artiodactyla	Impala	*Aepyceros melampus*	South Africa	47	11	CELISA	[28]
Mammalia	Artiodactyla	Klipspringer	*Oreotragus oreotragus*	South Africa, Zimbabwe	1	0	RPHI	[13]
Mammalia	Artiodactyla	Greater kudu	*Tragelaphus strepsiceros*	South Africa, Zimbabwe	78	21.8	RPHI	[13]
Mammalia	Artiodactyla	Greater kudu	*Tragelaphus strepsiceros*	South Africa	4	50	CELISA	[28]
Mammalia	Artiodactyla	Mountain reedbuck	*Redunca fulvorufula*	South Africa, Zimbabwe	3	33.3	RPHI	[13]
Mammalia	Artiodactyla	Nyala	*Tragelaphus angasii*	South Africa, Zimbabwe	5	40	RPHI	[13]
Mammalia	Artiodactyla	Nyala	*Tragelaphus angasii*	South Africa	1	0	CELISA	[28]
Mammalia	Artiodactyla	Red hartebeest	*Alcelaphus buselaphus*	South Africa, Zimbabwe	6	16.7	RPHI	[13]
Mammalia	Artiodactyla	Southern reedbuck	*Redunca arundinum*	South Africa, Zimbabwe	24	4.2	RPHI	[13]
Mammalia	Artiodactyla	Roan antelope	*Hippotragus equinus*	South Africa, Zimbabwe	2	0	RPHI	[13]
Mammalia	Artiodactyla	Roan antelope	*Hippotragus equinus*	South Africa	8	0	CELISA	[28]
Mammalia	Artiodactyla	Sable antelope	*Hippotragus niger*	South Africa	49	6	CELISA	[28]
Mammalia	Artiodactyla	Sable antelope	*Hippotragus niger*	South Africa, Zimbabwe	28	32.1	RPHI	[13]
Mammalia	Artiodactyla	Springbok	*Antidorcas marsupialis*	South Africa, Zimbabwe	69	1.4	RPHI	[13]
Mammalia	Artiodactyla	Steenbok	*Raphicerus campestris*	South Africa, Zimbabwe	12	0	RPHI	[13]
Mammalia	Artiodactyla	Suni	*Neotragus moschatus*	South Africa	4	0	CELISA	[28]
Mammalia	Artiodactyla	Common tsessebe	*Damaliscus lunatus*	South Africa	2	0	CELISA	[28]
Mammalia	Artiodactyla	Common tsessebe	*Damaliscus lunatus*	South Africa, Zimbabwe	1	0	RPHI	[13]
Mammalia	Artiodactyla	Warthog	*Phacochoerus aethiopicus*	South Africa, Zimbabwe	40	5	RPHI	[13]
Mammalia	Artiodactyla	Warthog	*Phacochoerus aethiopicus*	South Africa	21	0	CELISA	[28]
Mammalia	Artiodactyla	Waterbuck	*Kobus ellipsiprymnus*	South Africa, Zimbabwe	9	44.4	RPHI	[13]
Mammalia	Carnivora	Aardwolf	*Proteles cristatus*	South Africa, Zimbabwe	4	0	RPHI	[13]
Mammalia	Carnivora	Banded mongoose	*Mungos mungo*	South Africa, Zimbabwe	1	0	RPHI	[13]
Mammalia	Carnivora	Bat-eared fox	*Otocyon megalotis*	South Africa, Zimbabwe	10	0	RPHI	[13]
Mammalia	Carnivora	Black-backed jackal	*Canis mesomelas*	South Africa, Zimbabwe	6	0	RPHI	[13]
Mammalia	Carnivora	Cape fox	*Vulpes chama*	South Africa, Zimbabwe	1	0	RPHI	[13]
Mammalia	Carnivora	Cape grey mongoose	*Herpestes pulverulentus*	South Africa, Zimbabwe	1	0	RPHI	[13]
Mammalia	Carnivora	Caracal	*Felis caracal*	South Africa, Zimbabwe	17	0	RPHI	[13]
Mammalia	Carnivora	Cheetah	*Acinonyx jubatus*	South Africa, Zimbabwe	1	0	RPHI	[13]
Mammalia	Carnivora	Cheetah	*Acinonyx jubatus*	South Africa	14	0	CELISA	[28]
Mammalia	Carnivora	Clawless otter	*Aonyx capensis*	South Africa, Zimbabwe	1	0	RPHI	[13]
Mammalia	Carnivora	Fox	*Vulpes* spp.	Tajikistan (FRM Tajik SSR)	5	0	CF, AGDP	[76]
Mammalia	Carnivora	Common genet/small-spotted genet	*Genetta genetta*	South Africa	1	0	CELISA	[28]
Mammalia	Carnivora	Common genet/small-spotted genet	*Genetta genetta*	South Africa, Zimbabwe	10	0	RPHI	[13]
Mammalia	Carnivora	Genet	*Genetta g*. *senegalensis*	Senegal	NR	Positive		[73] in [6]
Mammalia	Carnivora	Honey badger	*Mellivora capensis*	South Africa, Zimbabwe	1	0	RPHI	[13]
Mammalia	Carnivora	Leopard	*Panthera pardus*	South Africa, Zimbabwe	1	0	RPHI	[13]
Mammalia	Carnivora	Leopard	*Panthera pardus*	South Africa	6	0	CELISA	[28]
Mammalia	Carnivora	African lion	*Panthera leo*	South Africa	116	0	CELISA	[28]
Mammalia	Carnivora	Red fox	*Vulpes vulpes*	Russia (Rostov Oblast)	5	40	IHI (neg by AGDP)	[102] in [6]
Mammalia	Carnivora	Red fox	*Vulpes vulpes*	Turkmenistan	NR	Positive	NR	[103] in [6]
Mammalia	Carnivora	Pallas’s cat	*Felis manul (*now *Otocolobus manul)*	Turkmenistan	NR	Positive	NR	[103] in [6]
Mammalia	Carnivora	Small spotted cat	*Felis nigripes*	South Africa, Zimbabwe	1	0	RPHI	[13]
Mammalia	Carnivora	Striped polecat	*Ictonyx striatus*	South Africa, Zimbabwe	5	0	RPHI	[13]
Mammalia	Carnivora	Suricate	*Suricata suricatta*	South Africa, Zimbabwe	3	33.3	RPHI	[13]
Mammalia	Carnivora	Water mongoose	*Atilax paludinosus*	South Africa, Zimbabwe	1	0	RPHI	[13]
Mammalia	Carnivora	African wildcat	*Felis lybica*	South Africa, Zimbabwe	3	0	RPHI	[13]
Mammalia	Carnivora	African wild dog	*Lycaon pictus*	South Africa	62	5	CELISA	[28]
Mammalia	Carnivora	Yellow mongoose	*Cynictis penicillata*	South Africa, Zimbabwe	7	0	RPHI	[13]
Mammalia	Cetartiodactyla	African buffalo	*Syncerus caffer*	South Africa, Zimbabwe	287	20	RPHI	[13]
Mammalia	Cetartiodactyla	African buffalo	*Syncerus caffer*	South Africa	312	10	CELISA	[28]
Mammalia	Chiroptera	Bats	Misc. spp.	France	19	15.3	AGDP	[104]
Mammalia	Chiroptera	Large mouse-eared bat	*Myotis blythii omari*	Iran	NR	Positive	AGDP	[9]
Mammalia	Chiroptera	Common noctule	*Nyctalus noctula*	Iran	NR	Positive	AGDP	[9]
Mammalia	Erinaceomorpha	Hedgehog	Misc spp.	Tajikistan (FRM Tajik SSR)	4	0	CF, AGDP	[76]
Mammalia	Erinaceomorpha	Long-eared hedgehog	*Hemiechinus auritus*	Turkmenistan (FRM Turkmen SSR)	NR	Positive	AGDP	[103]; [105] in [6]
Mammalia	Erinaceomorpha	South African hedgehog	*Erinaceus frontalis*	South Africa, Zimbabwe	8	0	RPHI	[13]
Mammalia	Hyracoidea	Rock hyrax	*Procavia capensis*	South Africa, Zimbabwe	19	0	RPHI	[13]
Mammalia	Insectivora	Dark-footed forest shrew	*Myosorex cafer*	South Africa, Zimbabwe	2	0	RPHI	[13]
Mammalia	Insectivora	Elephant shrew	*Elephantulus* spp.	South Africa, Zimbabwe	112	0	RPHI	[13]
Mammalia	Insectivora	Musk shrew	*Crocidura* spp.	South Africa, Zimbabwe	23	0	RPHI	[13]
Mammalia	Insectivora	Round-eared elephant shrew	*Macroscelides proboscideus*	South Africa, Zimbabwe	31	0	RPHI	[13]
Mammalia	Lagomorpha	Cape hare	*Lepus capensis*	South Africa, Zimbabwe	62	22.6	RPHI	[13]
Mammalia	Lagomorpha	Cape hare	*Lepus capensis*	Turkmenistan	NR	Positive	CF, AGDP	[103] in [6]
Mammalia	Lagomorpha	European hare	*Lepus europaeus*	Russia (Rostov Oblast)	20	20	IHI (neg by AGDP)	[102] in [6]
Mammalia	Lagomorpha	European hare	*Lepus europaeus*	Hungary	198	6	IgG ELISA, IFA	[23]
Mammalia	Lagomorpha	Greater red rock hare	*Pronolagus crassicaudatus*	South Africa, Zimbabwe	13	0	RPHI	[13]
Mammalia	Lagomorpha	Hare	*Lepus* spp.	South Africa, Zimbabwe	49	14.3	RPHI	[13]
Mammalia	Lagomorpha	Hare	*Lepus* spp.	South Africa	63	0	CELISA	[28]
Mammalia	Lagomorpha	Hare	*Lepus* spp.	Albania (Kukes)	4	0	IgG ELISA	[50]
Mammalia	Lagomorpha	Hare	*Lepus* spp.	Bulgaria	33	3	AGDP	[56]
Mammalia	Lagomorpha	Hare	*Lepus* spp.	Iran	NR	Positive	NR	[106] in [6]
Mammalia	Lagomorpha	Jameson’s red rock hare	*Pronolagus radensis*	South Africa, Zimbabwe	4	0	RPHI	[13]
Mammalia	Lagomorpha	Red rock hare	*Pronolagus* spp.	South Africa, Zimbabwe	9	0	RPHI	[13]
Mammalia	Lagomorpha	Scrub hare	*Lepus saxatilis*	South Africa, Zimbabwe	131	14.5	RPHI	[13]
Mammalia	Lagomorpha	Smith’s red rock hare	*Pronolagus rupestris*	South Africa, Zimbabwe	25	0	RPHI	[13]
Mammalia	Perissodactyla	Black rhinoceros	*Diceros bicornis*	South Africa	5	40	CELISA	[28]
Mammalia	Perissodactyla	Black rhinoceros	*Diceros bicornis*	South Africa, Zimbabwe	5	60	RPHI	[13]
Mammalia	Perissodactyla	Burchell’s zebra	*Equus burchelli*	South Africa, Zimbabwe	93	17	RPHI	[13]
Mammalia	Perissodactyla	White rhinoceros	*Ceratotherium simum*	South Africa, Zimbabwe	8	50	RPHI	[13]
Mammalia	Perissodactyla	White rhinoceros	*Ceratotherium simum*	South Africa	31	68	CELISA	[28]
Mammalia	Perissodactyla	Zebra	*Equus burchelli*	South Africa	28	7	CELISA	[28]
Mammalia	Primata	Chacma baboon	*Papio ursinus*	Kenya	226	0	AGDP	[77]
Mammalia	Primata	Chacma baboon	*Papio ursinus*	South Africa	21	0	CELISA	[28]
Mammalia	Primata	Chacma baboon	*Papio ursinus*	South Africa, Zimbabwe	289	0	RPHI	[13]
Mammalia	Primata	Vervet monkey	*Cercopithecus pygerythrus*	South Africa, Zimbabwe	233	0	RPHI	[13]
Mammalia	Primata	Vervet monkey	*Cercopithecus pygerythrus*	South Africa	1	0	CELISA	[28]
Mammalia	Proboscidea	African bush elephant	*Loxodonta africana*	South Africa, Zimbabwe	211	0.5	RHPI	[13]
Mammalia	Proboscidea	African bush elephant	*Loxodonta africana*	South Africa	23	0	CELISA	[28]
Mammalia	Rodentia	African marsh rat	*Dasymys incomtus*	South Africa, Zimbabwe	1	0	RPHI	[13]
Mammalia	Rodentia	Angoni vlei rat	*Otomys angoniensis*	South Africa, Zimbabwe	1	0	RPHI	[13]
Mammalia	Rodentia	Brown rat	*Rattus norvegicus*	South Africa, Zimbabwe	6	0	RPHI	[13]
Mammalia	Rodentia	Brown rat	*Rattus norvegicus*	Pakistan	9	22.2	CF	[43]
Mammalia	Rodentia	Karoo bush rat	*Otomys unisulcatus*	South Africa, Zimbabwe	52	0	RPHI	[13]
Mammalia	Rodentia	Bushveld gerbil	*Tatera leucogaster*	South Africa, Zimbabwe	61	9.8	RPHI	[13]
Mammalia	Rodentia	Cape ground squirrel	*Xerus inauris*	South Africa, Zimbabwe	37	2.7	RPHI	[13]
Mammalia	Rodentia	Coypu	*Myocastor coypus*	Tajikistan (FRM Tajik SSR)	156	0	CF, AGDP	[36,76]
Mammalia	Rodentia	Gerbil	*Meriones crassus*	Iran	NR	Positive	AGDP	[9]
Mammalia	Rodentia	Great gerbil	*Rhombomys opimus*	Turkmenistan (FRM Turkmen SSR, Bakharden region)	18	0	AGDP	[32]
Mammalia	Rodentia	Highveld gerbil	*Tatera brantsii*	South Africa, Zimbabwe	224	2.2	RPHI	[13]
Mammalia	Rodentia	House mouse	*Mus musculus*	South Africa, Zimbabwe	11	0	RPHI	[13]
Mammalia	Rodentia	House rat	*Rattus rattus*	South Africa, Zimbabwe	40	0	RPHI	[13]
Mammalia	Rodentia	House rat	*Rattus rattus*	Pakistan	54	1.9	CF	[43]
Mammalia	Rodentia	Indian bush rat	*Golunda ellioti*	Pakistan	1	0	CF	[43]
Mammalia	Rodentia	Indian bush rat	*Golunda ellioti*	Tajikistan (FRM Tajik SSR)	16	0	CF, AGDP	[36,76]
Mammalia	Rodentia	Indian desert jird	*Meriones hurrianae*	Pakistan	33	9	CF	[43]
Mammalia	Rodentia	Indian gerbil	*Tatera indica*	Pakistan	47	19	CF	[43]
Mammalia	Rodentia	Indian palm squirrel	*Funambulus pennanti*	Pakistan	2	0	CF	[43]
Mammalia	Rodentia	Lesser bandicoot rat	*Bandicota bengalensis*	Pakistan	2	0	CF	[43]
Mammalia	Rodentia	Libyan jird (red-tailed)	*Meriones libycus*	Tajikistan (FRM Tajik SSR)	4	0	CF, AGDP	[76]
Mammalia	Rodentia	Long-clawed ground squirrel	*Spermophilopsis leptodactylus*	Turkmenistan (FRM Turkmen SSR, Bakharden region)	1	0	AGDP	[32]
Mammalia	Rodentia	Long-tailed marmot	*Marmota caudata*	Tajikistan (FRM Tajik SSR, Murgab region)	288	0	AGDP	[36]
Mammalia	Rodentia	Long-tailed marmot	*Marmota caudata*	Tajikistan (FRM Tajik SSR, central)	275	0	AGDP	[36]
Mammalia	Rodentia	Misc. rodents		Iraq	35	14.2	CF	[35]
Mammalia	Rodentia	Misc. rodents		Iran	175	2.9	AGDP	[45]
Mammalia	Rodentia	Multimammate mouse	*Mastomys* spp. *(coucha*, *natalensis)*	South Africa, Zimbabwe	245	0.3	RPHI	[13]
Mammalia	Rodentia	Muskrat	*Ondatra zibethicus*	Tajikistan (FRM Tajik SSR, northern)	35	0	CF, AGDP	[36,76]
Mammalia	Rodentia	Namaqua gerbil	*Desmodillus auricularis*	South Africa, Zimbabwe	58	0	RPHI	[13]
Mammalia	Rodentia	Namaqua rock rat	*Aethomys namaquensis*	South Africa, Zimbabwe	95	1.1	RPHI	[13]
Mammalia	Rodentia	Cape porcupine	*Hystrix africaeaustralis*	Tajikistan (FRM Tajik SSR)	1	0	CF, AGDP	[76]
Mammalia	Rodentia	Cape porcupine	*Hystrix africaeaustralis*	South Africa, Zimbabwe	8	12.5	RPHI	[13]
Mammalia	Rodentia	Cape porcupine	*Hystrix africaeaustralis*	South Africa	2	0	CELISA	[28]
Mammalia	Rodentia	South African pouched mouse	*Saccostomus campestris*	South Africa, Zimbabwe	3	0	RPHI	[13]
Mammalia	Rodentia	South African springhare	*Pedetes capensis*	South Africa, Zimbabwe	33	12.1	RPHI	[13]
Mammalia	Rodentia	Pygmy mouse	*Mus minutoides*	South Africa, Zimbabwe	8	0	RPHI	[13]
Mammalia	Rodentia	Red veld rat	*Aethomys chrysophilus*	South Africa, Zimbabwe	35	0	RPHI	[13]
Mammalia	Rodentia	Short-tailed bandicoot rat	*Nesokia indica*	Pakistan	7	0	CF	[43]
Mammalia	Rodentia	Small five-toed jerboa	*Allactaga* spp.	Tajikistan (FRM Tajik SSR)	2	0	CF, AGDP	[76]
Mammalia	Rodentia	Griselda’s striped grass mouse	*Lemniscomys griselda*	South Africa, Zimbabwe	5	0	RPHI	[13]
Mammalia	Rodentia	Soft-furred rat	*Rattus (Millardia) meltada*	Pakistan	2	0	CF	[43]
Mammalia	Rodentia	Four-striped grass mouse	*Rhabdomys pumilio*	South Africa, Zimbabwe	344	0.6	RPHI	[13]
Mammalia	Rodentia	Acacia rat	*Thallomys paedulcus*	South Africa, Zimbabwe	2	0	RPHI	[13]
Mammalia	Rodentia	Turkestan rat	*Rattus pyctoris*	Tajikistan (FRM Tajik SSR)	8	0	CF, AGDP	[76]
Mammalia	Rodentia	Vlei rat	*Otomys irroratus*	South Africa, Zimbabwe	36	0	RPHI	[13]
Reptilia	Squamata	Blunt-nosed viper	*Macrovipera lebetina*	Tajikistan	1	0	CF, AGDP	[76]
Reptilia	Squamata	European legless lizard (sheltopusik)	*Pseudopus apodus*	Tajikistan (FRM Tajik SSR)	4 (or 5)	0	CF, AGDP	[36,76]
Reptilia	Testudinata	Horsfield’s tortoise	*Testudo horsfieldii*	Tajikistan	60	1.6%‡	AGDP	[6,36]

‡ Only known report of seropositive result in taxonomic order.

AGDP, agar gel diffusion precipitation; CELISA, competitive ELISA; CF, antibody complement fixation; IHI, indirect hemagglutination inhibition test; N/A, not applicable; NR, not reported; RPHI, reverse passive hemagglutination-inhibition assay; FRM, formerly; SSR, Soviet Socialist Republic.

Two reports have found antibodies to CCHFV in representatives of the mammalian order Chiroptera. Using the AGDP test with antigens prepared from CCHFV strains isolated in then-Soviet republics, antibodies were detected in blood sera from two bats in France, from an area bordering with Spain [104]. The species sampled were not specified, and this remains the only report of CCHFV seroprevalence in France. One additional study in northern Iran reported evidence by AGDP in Chiroptera species, in the sera of the greater mouse-eared bat and the common noctule [9]. While these reports appear to be the only evidence of CCHFV infection in bats, recent investigations into bat viruses suggest that there are other species of nairoviruses circulating in bat populations. Using modern sequencing techniques, the first bat nairovirus was identified in French insectivorous bat specimens [107], and a novel nairovirus was isolated from Zambian bats [108].

In reptiles, anti-CCHFV antibodies were detected in one Horsfield’s tortoise (*Testudo horsfieldii*) trapped in early June in Bul’yoni-Bolo winter camp in the Dangara region of Tajikistan [36]. There are several conflicting reports as to the total sample size of the study, ranging from four to 209 tortoises [6,36]; reported seroprevalence is based on the most detailed report provided by T.P. Pak [36]. Other limited investigations of reptile samples did not yield any evidence of antibodies to CCHFV [36,76]. However, a recent report detected CCHFV in *Hyalomma aegyptium* [109], the tortoise tick, suggesting that tortoises may be similar to certain bird species (discussed below), in which infected ticks are commonly found feeding on the animal, and CCHFV transmission to ticks may occur even in the absence of detectable antibodies in the host.

## Birds

Many bird species are important hosts for *Hyalomma* ticks and can transport ticks over long distances [110,111]. The transport of CCHFV-infected ticks by birds is a current topic of concern regarding regional spread of the virus [29,112]. Historical studies found birds associated with cattle pastures to be important in feeding immature tick stages, and that rooks (*Corvus frugilegus*) were particularly important; an increase in CCHF cases was associated with increased rook populations [113]. However, CCHFV infection and the presence or absence of an antibody response in avian species remains unclear. The majority of serosurveys of wild avian species report no serological evidence of CCHFV infection in birds, despite investigation of numerous species and substantial sample pools (Table 2). This absence of viremia is interesting, as some species support large numbers of CCHFV-infected ticks [6,69]. This observation has been supported by experimental infection; the red-billed hornbill (*Tockus erythrorhynchus*) was found to replicate CCHFV without detectable viremia and was able to infect immature *Hyalomma rufipes* ticks [114,115]. However, another experimental infection study of mostly ground-feeding birds suggested that anti-CCHFV antibodies may be produced following infection; blue-helmeted guineafowl (*Numida meleagris*), for example, developed low-level viremia followed by a transient antibody response [17]. Studies on Anseriformes and Galliformes species are also conflicting. In pathogenicity studies, experimentally infected domestic chickens were found to be refractory to CCHFV infection [17]. However, a 0.2% CCHFV seroprevalence in chickens and ducks (*n* = 428) was reported in Kazakhstan [66].

The absence of detectable anti-CCHFV antibodies in birds may reflect limitations in assay sensitivity. Most of the serological surveys on birds in the former USSR were based on the AGDP test [6], and several studies have shown that the AGDP test is less sensitive than the RPHI or IFA tests for detection of CCHFV antibodies [13,17,37]. More recent investigations, however, suggest that past reports accurately reflect the absence of antibody production, and that most species of birds do not appear to develop viremia. An investigation by Shepherd et al. on the sera of 460 birds of 37 species failed to detect antibodies to CCHFV [17]. However, the absence of antibody production is not universal to all bird species. Ostriches appear to be an exception amongst avian species in harboring and possibly transmitting CCHFV to humans. In the above-mentioned studies by Shepherd et al., anti-CCHFV antibodies were found in 22/92 (23.9%) ostriches (*Struthio camelus*). Of note, antibodies were detected in 6/9 (66.6%) ostriches in association with a human CCHF case in a worker who became ill after slaughtering ostriches on a farm in South Africa [17]. Additionally, 1/5 (20%) ostriches tested in association with four CCHF cases in workers from two ostrich farms in Iran were also found to be positive for CCHFV IgG [80]. Experimental infection has shown that viremia in ostriches is very short in duration [116].

## CCHFV Isolation from Animals

Experimental studies suggest that many animal species develop a transient viremia, and thus may play a role in transmitting CCHFV to ticks in nature. However, reports of CCHFV isolation from animals are limited. CCHFV has been isolated from a febrile cow in Kenya, cattle and a goat in a Nigerian abattoir, a goat placed as a sentinel for arboviruses in Senegal, European hares in Crimea, and a hedgehog in Nigeria (Table 3). Further supporting serological data, in an extensive study in endemic foci in Russia (Astrakhan Oblast), no virus was isolated from over 350 bird specimens representing 35 species.

**Table 3:** CCHFV isolation from domestic and wild animals.

Common name	Scientific name	Country of Origin	No. Isolates	Reference
**Cattle**	*Bos* spp.	Kenya (Nakaru)	1	[18]
		Nigeria	4	[117]
**European hare**	*Lepus europaeus*	Ukraine (Crimea)	3	[49]
**Goat**	*Capra* spp.	Nigeria	1	[117]
		Senegal (Bandia Forest)	1	[6,18]
**Hedgehog**	*Hemiechinus auritus*	Crimea	0/17	[118]
	*Erinaceus albiventris*	Nigeria	1	[117]
**Misc. birds**		Russia (Astrakhan Oblast)	0/360	In [6]

The paucity of CCHFV isolates from animals likely reflects a relatively brief viremic period and difficulty in identifying infected animals due to absent or mild clinical disease [119–121]. The majority of reported CCHFV isolations are from ticks or human case-patients. This is a result of an increased relative likelihood of isolation and, in turn, a preference for tick and human case specimens for isolation attempts. However, inability to isolate CCHFV from vertebrate animals does not necessarily indicate a lack of infection in these animals, and does not rule them out as potential CCHFV hosts capable of spreading disease to humans.

## Discussion

A large amount of research investigating the role of animals in transmission and maintenance of CCHFV was performed beginning in the late 1960s and 1970s. This work was instrumental in identifying mammalian species, particularly livestock, as critical in the maintenance of CCHFV and as sources of human exposure. The knowledge gained from these studies has also been important in developing prevention and control strategies such as the use of acaricides on livestock in endemic regions. Recently, numerous studies have provided additional information on known reservoir species and provided country-specific information on animal species with notable roles in CCHFV maintenance.

The reports summarized herein must be considered broadly and examined for trends and not specifics due to several factors. Reported seroprevalence may be biased by sample size, seasonality, and diversity in sampling sites, since if one animal is seropositive, additional positive animals are likely to be found in that location at that time. In addition, these reports used a variety of serological assays. There are caveats to interpretation of individual assay results [12], and direct comparison of results from a variety of assays is confounded by variation in assay sensitivity and specificity. Several groups have performed direct comparisons of the reported serological assays [20,36,69,122]; however, results of the comparisons themselves will vary depending on the conditions of the specific assay and the species investigated. Also, several iterations of the same format of serological tests have been used over the years, making generalized statements about their relative reliability challenging. Comparison of serological techniques for use in animals has been performed for other zoonotic viral hemorrhagic fevers [123]. For CCHFV, the merits and pitfalls of several of the serological assays were reviewed by Hoogstraal [6], who advises that most earlier seroepidemiological results be regarded as suggestive of CCHFV seropositivity but not as positive proof.

Overall, serological detection methods have improved over time. Technological advances, including the advent of ELISA assays, allow detection of low amounts of infectious virus or of inactivated antigen and antibodies to CCHFV, and have been shown to be more sensitive, specific, rapid, and reproducible than CF, IFA, RPHI, or AGDP [124]. ELISAs are generally considered the preferred method of serological investigation for CCHFV. However, sandwich ELISA techniques cannot be applied successfully to all species [28], necessitating further advances in testing, including a CELISA that was validated during an extensive CCHFV serological survey in South Africa [28]. Of note, species-specific validations of ELISAs have been performed; Qing et al. evaluated a recombinant nucleoprotein-based system for IgG detection in sheep sera [125], and Mertens et al. developed an ELISA for CCHFV IgG antibodies in bovine sera, showing it to have >98% diagnostic sensitivity and specificity [24].

Finally, there is also the potential for cross-reactivity with other related nairoviruses such as Dugbe virus, Nairobi sheep disease, and Qalyub viruses [20,25]. Antibodies to other nairoviruses may exist independently or in conjunction with CCHFV-specific antibodies. Thus, reports of seroprevalence in areas not previously identified to have CCHFV transmission would benefit from additional surveillance, such as tick studies, to help support novel identification of CCHFV foci.

Irrespective of the nuances of serological assay interpretation and incongruity, the data from the studies summarized here, importantly, indicate broad areas with endemic transmission and highlight reservoir species with the highest potential to affect public health. Some species may serve as direct sources of viral transmission (e.g., viremic livestock, ostriches), whereas others aid principally in maintaining high levels of CCHFV endemicity (e.g., hares). These data also highlight species that could present a risk but have not previously been implicated in human cases, such as camels that are replacing cattle use in certain regions due to climate change [126].

With extensive areas of endemic transmission, the issue of CCHFV importation via animal hosts, ticks, or human cases is a critical concern. Importation of livestock was highlighted in a 1994–1995 CCHFV outbreak in the United Arab Emirates; CCHFV sequences from the patients of this outbreak were identical or closely related to those from three *Hyalomma* spp. ticks obtained from livestock recently imported from Somalia [127]. It is not clear, however, whether the imported animals were infected at the time of importation or more susceptible to infection upon arrival. Williams et al. [46] reported higher seroprevalence in imported sheep and goats than in indigenous animals, which was attributed to increased susceptibility of naïve animals and virus circulation within the quarantine areas. A subset of the sheep sampled was from Western Australia, a region in which no CCHFV-competent vectors have been reported. The majority of imported animals surveyed from Australia had been in Oman for more than 30 days and, although reported as tick-free upon entry, had high levels of *Hyalomma* spp. infestation at the time of sampling, providing opportunity for CCHFV exposure. Importation of human cases has also occurred. To date, four human cases of CCHF have been imported into a non-endemic country: in 2004, a case was imported into France from Senegal [128]; in 2009, a US soldier entered Germany from Afghanistan; in 2012, an infected person arrived in the United Kingdom from Afghanistan; and in 2014, another came into the UK from Bulgaria. Other unconfirmed reports include a suspected case imported to the UK from Zimbabwe in 1997 and into Germany from Bulgaria in 2001 [129].

CCHFV is widely distributed, circulates in numerous vertebrate species, and can be transmitted to humans in several ways. Serosurveillance of animals will continue to be an essential tool for monitoring levels of endemic transmission and for investigating areas where CCHFV is not known to circulate. The importance of timely assessment of the potential role of domestic and wildlife species in disease introduction and emerging disease response is very important in the case of CCHFV. Our report summarizes data from international studies investigating the presence of antibodies to CCHFV in domestic and wild animals. We provide comprehensive species-specific information and highlight the appropriate literature serving as a critical resource in future discussion of putative importation and extension of known CCHFV endemicity.

**Key Learning Points**

- Anti-CCHFV antibodies are detected in a wide spectrum of domestic and wild animals from many countries.
- Cattle, followed by sheep and goats, have been investigated in the largest number of seroepidemiological studies.
- Despite a high tick burden in many avian species, anti-CCHFV antibodies have not been detected in birds, with the exception of guinea fowl and ostriches.
- Epidemiological evidence and serological data show that handling livestock species (i.e., cattle, sheep, goats, ostriches) can serve as a source of disease transmission to humans.
- CCHFV seroepidemiological data in animals is an indicator of potential disease foci.

**Top Five Papers**

- Causey OR, Kemp GE, Madbouly MH, David-West TS. Congo virus from domestic livestock, African hedgehog, and arthropods in Nigeria. Am J Trop Med Hyg. 1970;19(5): 846–50.
- Hoogstraal H. The epidemiology of tick-borne Crimean-Congo hemorrhagic fever in Asia, Europe, and Africa. J Med Entomol. 1979;15(4): 307–417.
- Donets M, Rezapkin G, Ivanov A, Tkachenko E. Immunosorbent assays for diagnosis of Crimean-Congo hemorrhagic fever (CCHF). Am J Trop Med Hyg. 1982;31: 156–62.
- Shepherd A, Swanepoel R, Leman P, Shepherd SP. Field and laboratory investigation of Crimean-Congo haemorrhagic fever virus (Nairovirus, family Bunyaviridae) infection in birds. Trans R Soc Trop Med Hyg. 1987;81: 1004–7.
- Shepherd AJ, Swanepoel R, Shepherd SP, McGillivray GM, Searle LA. Antibody to Crimean-Congo hemorrhagic fever virus in wild mammals from southern Africa. Am J Trop Med Hyg. 1987;36(1): 133–42. http://www.ncbi.nlm.nih.gov/pubmed/3101526
